# Prepartum and/or postpartum supplementation with monensin-molasses multinutrient blocks to optimize fertility and calf performance in primiparous beef cows

**DOI:** 10.5713/ab.22.0068

**Published:** 2022-05-02

**Authors:** Bruna Lima Chechin Catussi, Laísa Garcia da Silva, Fernando José Schalch Júnior, Rafaela Maria Sutiro Angelieri Auder, Juan Fernando Morales Gómez, Rodolfo Daniel Mingoti, Sérgio Carlos Franco Morgulis, Pietro Sampaio Baruselli

**Affiliations:** 1 Department of Animal Reproduction, Faculty of Veterinary Medicine and Animal Science, University of Sao Paulo, Sao Paulo, 05508-210, SP, Brazil; 2 Minerthal Nutritional Products, Sao Paulo, 01451-001, SP, Brazil; 3 Department of Animal Science, Faculty of Animal Science and Food Engineering, University of São Paulo, Pirassununga, 12635-900, SP, Brazil; 4 Mater farm, Santa Rita do Pardo, 79690-000, MS, Brazil

**Keywords:** Beef Cattle, Calves, Fixed Timed Artificial Insemination (FTAI), Nutrition, Pregnancy, Reproduction

## Abstract

**Objective:**

Pregnant Nelore heifers (n = 417) were used to evaluate the effects of supplementation with monensin-molasses multinutrient block (B) during pre and/or postpartum on reproductive and progeny performance.

**Methods:**

Heifers were allocated in four treatments: i) CC: heifers received control supplement (C) in loose meal form (0.06% of body weight [BW] offered daily before and after parturition; n = 108); ii) CB: received C before parturition and B (0.07% of BW offered weekly after parturition; n = 117); iii) BC: received B before and C after parturition (n = 103) and iv) BB: received B before and after parturition (n = 89). During pre and postpartum periods, concentration of metabolites/hormones and cow/calf performance was evaluated over time. Cows were synchronized twice for fixed timed artificial insemination (FTAI) using an estradiol/progesterone-based protocol. Data was analyzed by orthogonal contrasts (C).

**Results:**

B increased pregnancy at first FTAI (p = 0.04) and overall pregnancy rate (C1: CC vs BB+BC+CB; p = 0.05). Supplemented cows had greater body condition score (BCS) only at parturition (D0; p = 0.04) and at D40 (p = 0.02). B increased BW (p = 0.03), glucose concentrations (p = 0.01) and subcutaneous fat thickness (p = 0.03) only at D40. Concentrations of insulin were higher in supplemented cows (p = 0.008). Calves born by cows supplemented before and after parturition (C2: BB vs BC+CB) were heavier at 80 (p<0.001), 120 (p<0.001), 170 (p = 0.002) and 210 (p = 0.02) days old.

**Conclusion:**

Regardless of period of treatment, block supplementation increased pregnancy at first FTAI and overall pregnancy rate. Additionality, block supplementation during both pre and postpartum periods improved progeny weight until weaning. Block supplementation can be a tool to optimize fertility and calf performance in Nelore primiparous cows.

## INTRODUCTION

Nutritional management is considered one of the most important factors that affect reproduction of beef cattle [1,2]. Brazilian commercial farms have the highest concentration of births in the dry season and during the transition to the rainy season. Consequently, beef cows spend most of their peripartum period with a low-quality forage available [3], which may lead to an inadequate intake of nutrients [4,5].

The higher gestational energetic/protein demand in the last third of gestation plus inadequate nutrient intake results in low body condition score (BCS) at parturition and negative energy balance in early postpartum [6]. As already noted, low energy reserves compromise the postpartum anestrous interval [7,8] and pregnancy rates in beef cows submitted to fixed timed artificial insemination (FTAI) [9]. This relationship is especially critical for primiparous cows, due to the additional demands needed to continue their own growth combined with the stress of first lactation [10]. Thus, inadequate nutrient intake before and/or after calving has greater detrimental effects on reproductive performance in primiparous than mature cows [6,11,12].

Supplementation programs for beef cows during prepartum and/or postpartum can be a useful strategy for improving the nutritional efficiency in grazing systems, especially when protein supplements are used [13]. Protein supplements can improve the activity of the rumen microbiota and fiber degradation, allowing for a better utilization of the forages, particularly during the dry season [14,15]. Studies have shown that supplemental protein for beef cattle grazing low-quality forage has a positive effect on increasing forage intake, body weight (BW), BCS, blood metabolites and reproductive efficiency [13,16]. Furthermore, strategies to increase blood glucose and insulin concentrations, such as feeding monensin, could enhance reproductive outcomes [17,18].

However, nutritional supplementation programs considerably increase production costs in beef cattle systems, such as the vehicle maintenance, fuel and labor costs that are required for daily supplemental feeding [11]. Block supplements consist of molasses, with ingredients that supply nutrients such as protein, minerals, vitamins, and additives [19]. These blocks have unique characteristics that limit the intake and can therefore be delivered less frequently (once a week or every 10 days) while avoiding overconsumption. Furthermore, molasses blocks improve forage intake, digestion, and the grazing of underutilized pastures, proving to be a potential strategy to decrease production costs and better the nutritional status of beef cattle [20–22].

Thus, we hypothesized that supplementation with monensin-molasses multinutrient block offered weekly improves the reproductive efficiency of primiparous beef cows and their progeny performance. The current study aimed to evaluate the effects of block supplementation during pre (90 days before calving) and/or postpartum (120 days after calving) periods on pregnancy rate and metabolic/hormonal characteristics of grazing primiparous Nelore cows as well as their progeny growth.

## MATERIALS AND METHODS

The experiment was conducted in a commercial farm located in Santa Rita do Pardo, Mato Grosso do Sul, Brazil, from June 2018 to May 2019. All animal-related procedures used in this study were approved by the Ethics Committee on Animal Use of the School of Veterinary Medicine and Animal Science (University of São Paulo, Brazil) under the protocol number 8169050819.

### Animals, experimental design and treatments

A total of 417 Nelore (*Bos indicus*) heifers in the final trimester of their pregnancy were assigned to this experiment. Heifers were 31.6±2.3 months of age (mean±standard error), weighed 438.8±3.5 kg and BCS (1 to 5 scale) of 2.94±0.03 at the beginning of the supplementation period (D-90). Treatments consisted of control supplementation (C: mineral supplement in loose meal form; 0.06% of BW; offered daily) used routinely on the farm, and molasses-monensin block supplementation (B: mineral protein supplement in block form; 0.07% of BW/d; offered weekly) recommended by Minerthal Nutritional Products Ltd (São Paulo, Brazil), offered in the proportion of 1 block (25 kg) for 10 cows (Table 1). A quantity of supplements was chosen to meet the protein requirements of primiparous cows, according to recommendations of Nutrient Requirements of Zebu and Crossbred Cattle [23].

**Figure 1:**
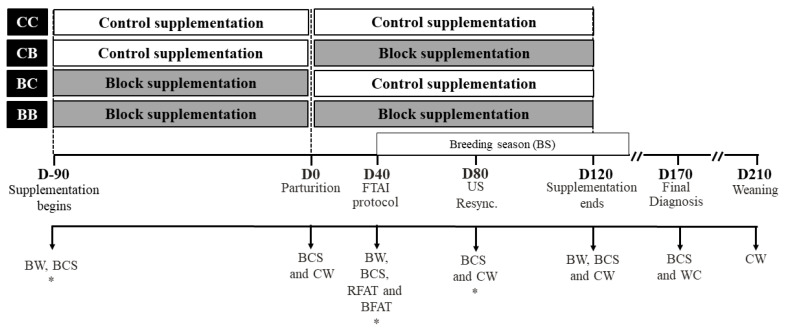
Experimental timeline. Ninety days before parturition (D-90) initiated the supplementation period, heifers were evaluated BW, BCS and blood sample (*) was collected (subset). At parturition calves were weighed and BCS of cows were evaluated. Forty days after calving (D40), cows were evaluated BW, BCS, RFAT, and BFAT. At the same time, cows were synchronized to FTAI and blood samples were collected (subset). Eighty days after calving (D80), blood sample was collected (subset), BCS and pregnancy diagnosis was performed. Non-pregnant cows were resynchronized to a second FTAI and CW was evaluated. A second pregnancy diagnosis was performed (D120) and BCS, BW and CW were evaluated. A final diagnosis for overall pregnancy rate (1st+2nd+NM) was performed on D170, and BCS and CW were evaluated. At the weaning (D210), all the calves were weighed. BW, body weight; BCS, body condition score; RFAT, subcutaneous rump fat thickness; BFAT, subcutaneous backfat thickness; FTAI, fixed timed artificial insemination; CW, calf body weight.

At the start of the supplementation period (90 days before the expected date of parturition) heifers were randomly distributed (according to predicted calving date, BW and BCS), as seen in Figure 1, in 4 treatments: i) Group CC: heifers received C 90 days before and 120 days after calving (n = 108); ii) Group CB: heifers received C 90 days before and B 120 days after calving (n = 117); iii) Group BC: heifers received B 90 days before and C 120 days after calving (n = 103); and iv) Group BB: heifers received B 90 days before and 120 after calving (n = 89). The cows were distributed in 4 paddocks, 2 groups of cows received B and 2 groups received C. Total supplementation period was 210 days: 90 days before parturition until the second pregnancy check (D120). After the supplementation period, all cows received C until the end of the breeding season (BS).

**Figure 2:**
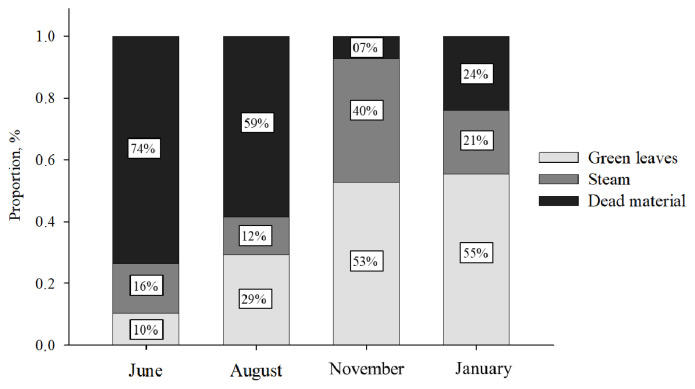
Botanic composition of pastures (8 paddocks/39 ha each) over the time.

During the trial period, the animals were kept in rotational grazing systems containing 8 paddocks of 39 hectares each. The groups of cows were rotated through the grazing systems every 7 days to avoid the effect of variation among pastures. The pasture was composed of grasses of the genus Urochloa brizantha. All the paddocks had a collective feeder, which provided at least 20 cm of linear feeder space per animal, avoiding competition between animals. Prior to grazing, forage samples were collected by hand-plucked sampling four times: June (Beginning of supplementation), August, November, and January (end of supplementation). Samples were cut at the ground level from five delimited areas (0.5× 0.5 m), selected randomly in each paddock to quantify herbage mass (Table 2). The green leaves were separated from the other structural components of the pasture (stem and dead material), weighed, pre-dried in a forced air oven at 55°C for 72 hours, and ground in a Willey mill with a 1-mm sieve. The mean chemical analysis of the green leaves is presented in Table 2, and the botanic composition of pastures in Figure 2.

### Ovulation synchronization protocol for fixed timed artificial insemination

Cows were assigned to FTAI protocol at 40.7±7.8 days postpartum. In a random day of the estrous cycle (day 0 of protocol), all cows received an intravaginal device with 0.6 g of progesterone (P4; Fertilcare 600, MDS, Brazil) and 2.0 mg intramuscular injection of estradiol benzoate (Fertilcare sincronização, MSD, Brazil). At the same time, the cows were classified as cyclic if they had a *corpus luteum* (CL) detected by an ultrasonography exam (DP-2200 VET; Mindray, Shenzhen, China). Eight days later (day 8 of protocol), the P4 device was removed, and 0.530 mg of sodic cloprostenol (Ciosin, MSD, Brazil), 1 mg of estradiol cypionate (Fertilcare ovulação, MSD, Brazil), and 300 IU of equine chorionic gonadotropin (Folligon, MSD, Brazil) was given by intramuscular injection. The cows were inseminated 48 hours after P4 device removal (day 10 of protocol), by the same technician, using two semen batches from a previously tested bull. The semen batches were homogeneously distributed between the treatments.

Pregnancy diagnosis was performed by transrectal ultrasonography 30 days after the first AI artificial insemination (AI; D80). Non-pregnant cows were resynchronized to a second FTAI using the same hormonal protocol described above. Fifteen days after the second FTAI, all the cows were exposed to natural mating with clean-up bulls at a proportion of 1 bull: 20 cows until the end of the BS (D140). Thirty days after the second FTAI (D120), the resynchronized cows had their pregnancy diagnostic. All animals were examined by transrectal ultrasonography 30 after the end of the BS (D170) to determine pregnancy status and pregnancy loss.

### Cow and calf performance

All animals had their BCS evaluated at five different times during the experimental period: D-90 (before parturition); D0 (parturition); D40; D80 and D120. The BCS attributed to each animal was performed using the visual technique [24] by the same trained technician. Animals were classified using a 1 (very thin) to 5 (very fat) point scale, with a difference of 0.25 points from one class to the next. Furthermore, cows were weighed at D-90; D40 and D120, respectively. The cows BW was obtained using a digital balance which all the animals were weighed individually at the same time of day. The calves had their body weight (CW) evaluated on D0 (at birth), D80, D120 (end of the supplementation), D170, and D210 (at weaning) to estimate growth and weight gain.

### Subcutaneous fat thickness evaluation

Forty days after parturition, at the onset of FTAI protocol (D40), subcutaneous backfat thickness (BFAT) and subcutaneous rump fat thickness (RFAT) were measured in all the animals. Ultrasound measurements were taken with an Aloka 500 SV (Hitachi Aloka Medical America, Inc., Wallingford, CT, USA) instrument equipped with a 3.5-MHz 172-mm linear transducer. Measurements of BFAT were taken in a transverse orientation between the 12th and 13th ribs approximately 10 cm distal from the midline. To RFAT the transducer was linearly positioned between hooks and pins at the sacral examination site and moved slightly until the correct image was formed, allowing for the visualization of the superior limit of the biceps femuris muscles. Ultrasound images were processed using Lince software (M & S Consultoria Agropecuária Ltda., Pirassununga, Brazil).

### Blood sampling, metabolites and hormone determinations

Blood samples were taken over time on a subset of cows (n = 120), using a tube through the coccygeal vein/artery. Blood samples were collected to measure glucose and urea concentrations during postpartum (D40 and D80), using a Vacutainer tube containing ethylenediaminetetraacetic acid (EDTA) and sodium fluoride (BD Vacutainer Fluoreto/EDTA, São Paulo, Brazil). A second blood sample were collected in a tube containing gel for serum separation and clot activation (BD Vacuntainer SST II Plus, Brazil) to analyze insulin-like growth factor-1 (IGF-I) and insulin concentration during prepartum (D-90), and postpartum (D40 and D80). Centrifugation of both tubes (2,000×g for 20 min) was performed to separate plasma and serum. Plasma/serum were removed and stored at −20°C for further analysis.

Serum concentrations of insulin were measured via commercial RIA kit (Sigma, St. Louis, MO, USA). Intra- and inter assay coefficients of variation (CV) were 9.9% and 14.9%. Serum concentrations of IGF-I were analyzed using in house competitive enzyme-linked immunosorbent assays (cELISA; Ansh labs, Webster, TX, USA) for bovine with the amplification biotin-streptavidin peroxidase system. Intra- and inter assay CV were 7.4% and 11.0%. Commercial enzymatic-colorimetric kits were used to determine plasma concentrations of glucose (K0827; Bioclin, Belo Horizonte, Brazil) and urea (K047; Bioclin, Brazil). Plasma urea nitrogen was estimated as 46.67% of the total plasma urea.

### Statistical analysis

The experiment followed a randomized complete block design, with random effect of group (group of cows/calf in a pasture in which treatment was applied) nested within treatment identifies the group as the experimental unit.

Distributions of the residuals of continuous data, such as cow and calf performance and metabolites and hormone profile, were evaluated for normality using graphical diagnostics, and data transformation was performed when appropriate. Variables that did not follow these assumptions were transformed accordingly and outliers were removed when necessary. Data were analyzed by the GLIMMIX procedure of SAS (SAS/STAT ver. 9.4) using the following model adapted to St-Pierre, 2007:


$$Y_{ijk}=\mu +D_{i}+T_{j}+D_{i}\times T_{j}+a_{k}+{\text{B}}_{\text{x}}+e_{ij},$$

where *Y_ijk_* = dependent variable; *μ* = overall mean; *D_i_* = fixed effect of treatment; *T_j_* = random effect of time; *D_i_*×*T_j_* = interaction between treatment and time; *a_k_* = random effect of animal within group of animals; β_x_ is the covariate adjustment for each animal; and *e_ijk_* = residual error.

Unstructured method was used to calculate the covariance structure. The Kenward-Roger method was used to calculate the denominator degrees of freedom. Unstructured UN(1) was the best covariance structure based on the smallest Akaike’s information criterion values. Other covariance structures were tested including compound symmetry, heterogeneous compound symmetry, first-order autoregressive and heterogeneous autoregressive. In addition, the data from the first sampling date of BW, BCS, CW, insulin and IGF-1 were added as a covariate in the statistical model.

Contrasts were constructed to evaluate the treatments: block supplementation effects (contrast 1 [C1]: Control×Block supplementation [CC×BB+BC+CB]); Block supplementation effects in both pre and postpartum periods (contrast 2 [C2]: pre and postpartum×pre or postpartum [BB×BC+CB]); and Block supplementation effects only during prepartum or only during postpartum (contrast 3 [C3]: prepartum× postpartum [BC×CB]). Data are presented as means±standard error of the mean, obtained using PROC MEANS of SAS. Statistical significance was defined as p≤0.05.

Variables with a binomial distribution, such as cyclicity rate, pregnancy per AI (P/AI), pregnancy per natural breeding and pregnancy loss, were analyzed by logistic regression using GLIMMIX procedure (SAS/STAT ver. 9.4). Initial models contained the following categorical explanatory variables as fixed effects: treatment, cyclic status (cyclic or noncyclic), AI technician, sire, straw(sire), BCS change (gained, maintained, or lost) and their first order interaction. Animal within group of animals were included as random effects in the model. Selection of the fixed effects model that best fit the data for each variable of interest was performed by finding the model with the lowest value for the Akaike information criterion using a backward elimination procedure that sequentially removed all variables with p≥0.10 from the model. Final models included the fixed effects of treatment and the random effects of animal within group of animals. The same contrasts were constructed to evaluate the treatments. Statistical significance was defined as p≤0.05.

## RESULTS

### Reproductive performance

**Figure 3:**
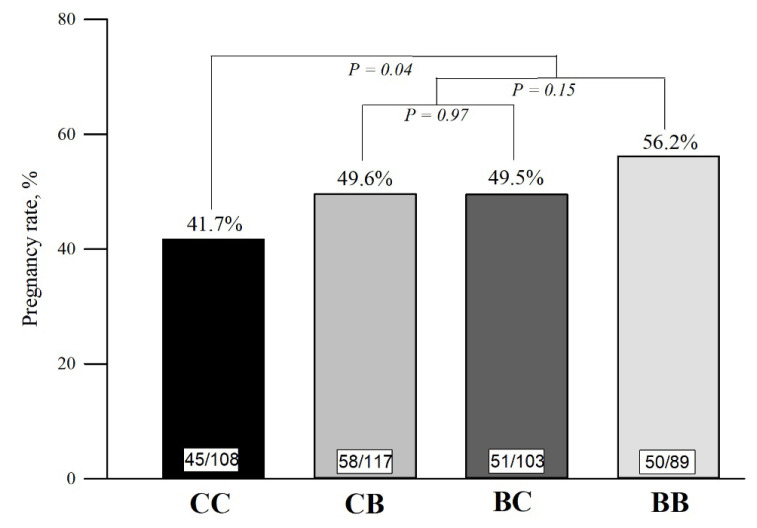
Pregnancy rate (%) at the first FTAI according to treatments in primiparous Nelore cows. Orthogonal contrasts: C1 (Block supplementation effect): Control (CC) vs block supplementation (BB+BC+CB); C2 (Block supplementation effect on pre and postpartum): Pre and postpartum (BB) vs Pre or postpartum (BC+CB); C3 (Pre or postpartum effect): prepartum (BC) vs postpartum (CB).

The reproductive performance according to treatments is presented in Table 3. The pregnancy rate at first FTAI was 9.8 percentage points greater for cows treated with blocks (Block supplementation: 51.5% [159/309] vs Control: 41.7% [45/108]; *P_C1_* = 0.04), compared to control cows (Figure 3). Moreover, there was a block supplementation effect on the overall pregnancy rate at end of the BS, after two consecutive FTAI’s and NM (Block supplementation: 84.1% [260/309] vs Control: 76.9% [83/108]; *P_C1_* = 0.05). Despite the evident positive effects of block supplementation on the pregnancy rate at first FTAI and at the end of the BS, no differences for cyclicity (*P_C1_* = 0.39), pregnancy at second FTAI (*P_C1_* = 0.70), or pregnancy at NM (*P_C1_* = 0.28) were observed.

The block supplementation during pre and postpartum periods (C2) did not affect the cyclicity rate (*P_C2_* = 0.51), pregnancy at first (*P_C2_* = 0.15) and second FTAI (*P_C2_* = 0.93), pregnancy at NM (*P_C2_* = 0.72), or overall pregnancy rate (*P_C2_* = 0.54), when compared with cows supplemented only during pre or postpartum. Likewise, no reproductive differences were observed for C3. The cyclicity (*P_C3_* = 0.97), pregnancy at first (*P_C3_* = 0.97) and second FTAI (*P_C3_* = 0.95), pregnancy at NM (*P_C3_* = 0.51), and overall pregnancy rate at end of the BS (*P_C3_* = 0.81) were similar between cows supplemented only in prepartum compared with cows supplemented only in postpartum. The pregnancy loss after the first and second FTAI did not differ between any contrast (p≥0.3).

### Cow and calf performance

There was interaction between time and treatment for BW (p<0.0001; Table 4), BCS (p<0.0001; Table 4) and CW (p< 0.0001; Table 5). Supplemented cows (C1) had greater BW only at D40 (Block supplementation: 408.7±1.99 vs Control: 400.8±3.25 kg; *P_C1_* = 0.03), but not at D-90 (*P_C1_* = 0.83) or D120 (*P_C1_* = 0.16). A greater BCS at parturition (Block supplementation: 3.06±0.01 vs Control: 3.00±0.03; *P_C1_* = 0.02) and at D40 (Block supplementation: 2.91±0.02 vs Control: 2.83±0.03; *P_C1_* = 0.01) was found for supplemented cows compared to control cows; However, no effects for BCS at D-90 (*P_C1_* = 0.15), D120 (*P_C1_* = 0.56) or D170 (*P_C1_* = 0.34) were found among groups.

Calves body weight was higher for calves born by cows treated with block supplementation compared to those born by cows from the control group at birth (Block supplementation: 35.5±0.24 vs Control: 34.6±0.42 kg; *P_C1_* = 0.01), at D80 (Block supplementation: 101.3±0.91 vs Control: 97.8±1.37 kg; *P_C1_* = 0.03) and at D120 (Block supplementation: 132.6±1.05 vs Control: 123.9±1.59 kg; *P_C1_*<0.01). Nevertheless, there was no difference for CW at D170 (*P_C1_* = 0.55) and at weaning (*P_C1_* = 0.38; Table 5).

**Figure 4:**
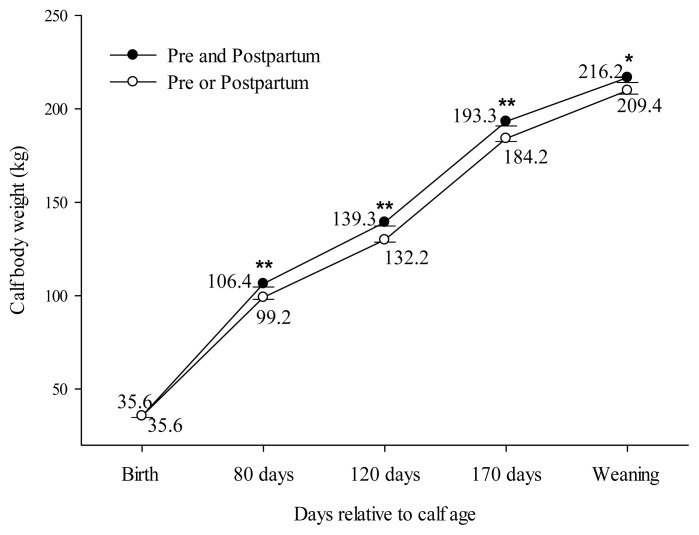
Effect of block supplementation during pre and postpartum period on calf body weight evaluated over the time. C2 (Block supplementation effect in both pre and postpartum periods): Pre and postpartum (BB) vs Pre or postpartum (BC+CB). ** Indicate a difference (p≤0.01) and * indicate a difference (p≤0.05).

Comparing block supplementation during pre and postpartum vs either pre or postpartum (C2), effect for BCS and CW was observed. However, no effect was found for BW. Cows treated during both pre and postpartum showed higher BCS only at D80 compared to cows treated during either pre or postpartum (Pre and postpartum: 2.98±0.04 vs Pre or postpartum: 2.88±0.02; *P_C2_* = 0.02). No effect was observed at D-90 (*P_C2_* = 0.45), D0 (*P_C2_* = 0.25), D40 (*P_C2_* = 0.21), D120 (*P_C2_* = 0.65) or D170 (*P_C2_* = 0.33) for BCS. The calves’ performance for C2 is presented in Figure 4. Calves born by cows supplemented with blocks during pre and postpartum showed the same birth weight as calves born by cows supplemented only in pre or postpartum (*P_C2_* = 0.89). However, CW was greater at 80 (Pre and postpartum: 106.4±1.74 vs Pre or postpartum: 99.2±1.04; *P_C2_*<0.001), 120 (Pre and postpartum: 139.3±1.97 vs Pre or postpartum: 129.9±1.20; *P_C2_*<0.001), 170 (Pre and postpartum: 193.3±2.44 vs Pre or postpartum: 184.2±1.57; *P_C2_* = 0.002) and 210 (Pre and postpartum: 216.2±2.03 vs Pre or postpartum: 209.4±1.48; *P_C2_* = 0.02) days old for calves born by cows treated with blocks during both pre and postpartum.

Block supplementation only during prepartum vs only during postpartum (C3) showed effects for BW, BCS, and CW. Cows supplemented during postpartum period had greater BW at D40 (Prepartum: 400.9±3.10 vs postpartum: 412.2±3.46; *P_C3_*<0.001), but not at D-90 (*P_C3_* = 0.18) or D120 (*P_C3_* = 0.11). The BCS at parturition was higher in cows treated during prepartum than cows treated during postpartum (Prepartum: 3.11±0.01 vs postpartum: 3.00±0.01; *P_C3_*<0.01). However, at the end of the BS (D170), cows treated in postpartum had greater BCS (Prepartum: 2.74±0.02 vs postpartum: 2.87±0.01; *P_C3_* = 0.001).

No effect of block supplementation was observed among the groups for CW at birth (*P_C3_* = 0.16), 80 (*P_C3_* = 0.75), 170 (*P_C3_* = 0.93), or 210 days old (*P_C3_* = 0.74). However, at the end of the supplementation period (D120), calves born by cows supplemented only during postpartum had greater CW when compared to calves born by cows treated only during prepartum (Prepartum: 126.5±1.65 vs postpartum: 132.9±1.69 kg; *P_C3_* = 0.002; Table 5).

### Subcutaneous fat thickness

**Figure 5:**
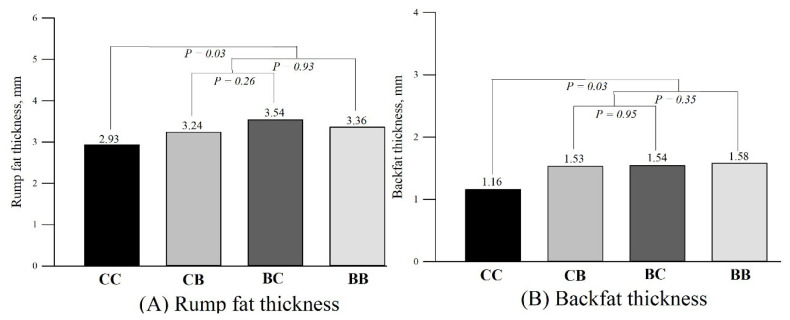
Subcutaneous fat thickness (mm) 40 days after parturition according to groups. Subcutaneous rump fat thickness (RFAT; A) and subcutaneous backfat thickness (BFAT; B). Orthogonal contrasts: C1 (Block supplementation effect): Control (CC) vs block supplementation (BB+ BC+CB); C2 (Block supplementation effect in both pre and postpartum periods): Pre and postpartum (BB) vs Pre or postpartum (BC+CB); and C3 (Pre or postpartum effect): prepartum (BC) vs postpartum (CB).

The subcutaneous fat thickness according to each group is presented in Figure 5. The rump fat thickness was greater for cows supplemented with blocks than control cows (3.36±0.10 vs 2.93±0.17 mm; *P_C1_* = 0.03). Likewise, there was a block supplementation effect for backfat thickness (Block supplementation: 1.58±0.07 vs Control: 1.16±0.16 mm; *P_C1_* = 0.03). The block supplementation during pre and postpartum (C2) did not affect RFAT (*P_C2_* = 0.93) and BFAT (*P_C2_* = 0.35). Additionally, there were no differences for RFAT (*P_C3_* = 0.26) and BFAT (*P_C3_* = 0.95) in cows supplemented only during pre or postpartum (C3).

### Metabolites and hormone profile

**Figure 6:**
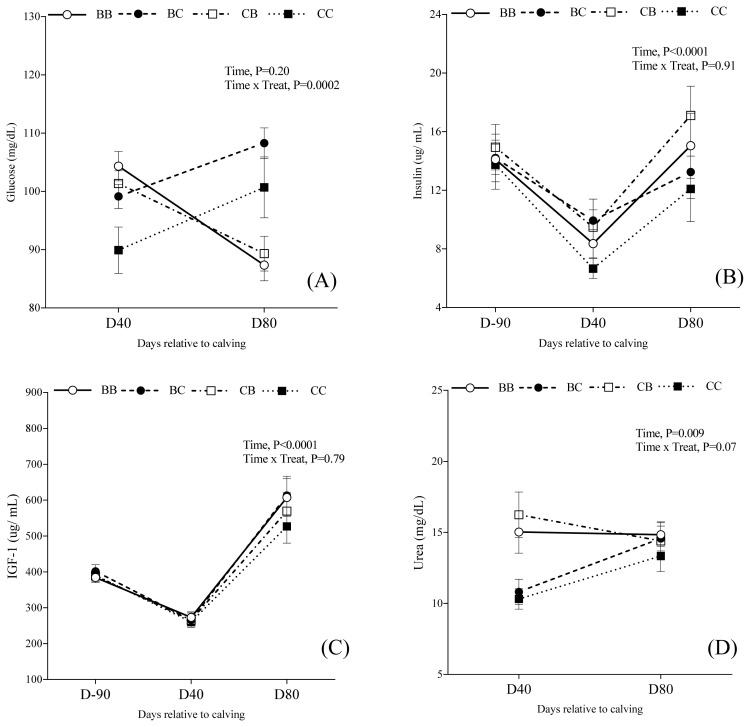
Effect of prepartum and/or postpartum supplementation with blocks on glucose plasma concentration (A), insulin serum concentration (B), IGF-1 serum concentration (C), and urea plasma concentration (D) of primiparous Nelore cows evaluated at different times. IGF-1, insulin-like growth factor-1.

The metabolite and hormone concentrations over time are presented in Table 6 and Figure 6, according to each group. There was an interaction between time and treatment for glucose concentration (p = 0.002), and urea concentration (p = 0.07), but not for insulin (p = 0.91) and IGF-1 (p = 0.79) concentration. Block supplementation effect (C1) was observed for glucose at D40 (Block supplementation = 101.5±1.9 vs Control = 89.9±4.9 mg/dL; *P_C1_* = 0.01), but not at D80 (*P_C1_* = 0.15). Likewise, the urea concentration was higher for supplemented cows when compared with control cows at D40 (10.31±0.7 vs 14.03±0.8 mg/mL; *P_C1_* = 0.03), but not at D80 (*P_C1_* = 0.41). Insulin serum concentration was higher for cows supplemented with blocks (*P_C1_* = 0.008). However, no effect for IGF-1 concentration was observed among groups (*P_C1_* = 0.24).

Cows supplemented with blocks during both pre and postpartum did not differ for glucose (*P_C2_* = 0.76), insulin (*P_C2_* = 0.13), IGF-1 (*P_C2_* = 0.35) or urea concentrations (*P_C2_* = 0.08) compared to cows supplemented during either pre or postpartum (C2). Furthermore, there was no effect of block supplementation in only pre or postpartum (C3) for insulin (*P_C3_* = 0.26) or IGF-I (*P_C3_* = 0.99) concentrations. Nevertheless, plasma concentration of glucose at D80 was greater in cows supplemented only during postpartum (Prepartum = 89.3±6.7 mg/dL vs Postpartum = 108.3±5.6; *P_C3_* = 0.03), but no effect was observed at D40 (*P_C3_* = 0.65). The urea concentration at D40 was higher in cows that were only supplemented during postpartum (Prepartum = 10.81±0.9 vs Postpartum = 16.24±1.6 mg/dL; *P_C3_* = 0.004), but not at D80 (*P_C3_* = 0.91).

## DISCUSSION

In the present study, supplementation with monensin-molasses multinutrient blocks increased BCS at parturition, improved the pregnancy at first FTAI, as well as the overall pregnancy rate at the end of the BS. Furthermore, cows supplemented with blocks had greater BW, BCS and a high index of subcutaneous backfat and rump fat thickness at the onset of the synchronization protocol, confirming our initial hypothesis. Several studies have shown a positive relationship between high BCS at parturition and fertility [8,9,25,26]. Additionally, other authors have demonstrated a strong correlation between subcutaneous fat index and the amount of fat in the carcass [24,27]. Body energy reserves are an essential source of readily available energy for reproduction, and beef cows had a greater likelihood of conceiving postpartum if they had greater BCS and rump fat thickness at parturition and during postpartum [1,2,8,9].

Nutrition impacts reproduction in beef cows through various changes in metabolic hormones [16]. Blood glucose can be used as measures of the energy status, and it is the primary fuel source used by the central nervous system which plays a major role in the release of gonadotropin-releasing hormone (GnRH) [8,15,26]. In the present study, the glucose concentration was higher for cows supplemented with blocks 40 days postpartum.

The composition of the block used in this study contains sodium monensin. Ionophores as monensin has been used in supplementation programs to graze ruminants, especially with low-quality forages [28]. Monensin alters the fermentation products of rumen microbes, increasing the production of propionate and decrease the acetate/propionate ratio, improving dry matter intake and protein digestibility, and increasing gluconeogenesis and glucose turnover [29]. Researchers reported that monensin supplementation decreases the interval between parturition and first estrus in beef cows [18] and increases dominant follicle diameter in beef heifers [30], as well as in postpartum Nelore cows [31]. Due to the experimental design, it is important to mention that the exact effect of monensin is not fully clear as there could be other factors affecting the results, such as the form of supplementation (Block vs loose meal). Bailey and Welling [20] reported that cows supplemented with low-moisture blocks visited the supplement more when compared with cows supplemented with conventional dry mixes, suggesting that blocks could modify ingestion and grazing patterns. The greater glucose concentration found during the postpartum period might be associated to the monensin treatment and the form of block supplementation.

Insulin and IGF-I synthesis are directly influenced by energy intake and circulating glucose concentrations [26,32]. Insulin concentration was higher for cows supplemented with blocks. Insulin is a mediator of nutritional effects on follicular dynamics in cattle, stimulating cell proliferation and steroidogenesis. Also, insulin may stimulate the release of GnRH from the hypothalamus [33]. Wettemann et al [10] have found that plasma concentrations of insulin, IGF-I, and glucose are associated with resumption of ovarian activity in postpartum beef cows, although no differences for IGF-I concentration was found in the present study.

All the changes in the endocrine and metabolic profile in cows supplemented with blocks may explain the higher pregnancy rate in the first TAI at the early postpartum (Figure 3). For pasture-based systems, high pregnancy rates in the beginning of the BS are critical for herd profitability [34]. According to Baruselli et al [35], cows exposed to FTAI at the beginning of the BS calved earlier, weaning heavier calves, and had improved probability of conceiving again in the subsequent BS.

A greater overall pregnancy rate at the end of BS was verified in primiparous cows supplemented with blocks, which means more calves would be born in the next season. In addition to the increase in reproductive performance, cows supplemented with blocks also presented good BCS at parturition and during the postpartum period. Moreover, calves born by cows supplemented with blocks were heavier from 80 to 170 days old (end of supplementation period = 120 days). Previous studies reinforced that the cows with good BCS at calving tend to wean heavier and healthier calves and this has important implications for young heifers destined to become breeders [16,36].

Supplementation with blocks during pre and postpartum did not promote sufficient changes on reproductive performance of primiparous cows (C2). However, cows supplemented during pre and postpartum had greater BCS at 80 days postpartum than cows supplemented in only one period. Improving maternal nutrition during postpartum improves milk yield and the higher calf nutrient intake acts upon the somatotropic axis, increasing calf growth and weight at weaning [37]. As a result, CW was higher until weaning for calves born by cows treated before and after parturition (Figure 4). This data corroborates with other authors that have shown a positive effect on calf growth at weaning when cows were supplemented during both pre and postpartum periods [7]. The focus for beef cow supplementation is usually to improve reproductive functions, but the enhancement of the nutritional status of beef cow diets may also influence the development of the future calf [38].

When comparing block supplementation in only pre and only postpartum periods (C3), a positive effect for block supplementation in prepartum was observed for BCS at parturition. However, 170 days after parturition cows supplemented in postpartum showed greater BCS. Primiparous cows appear to be more sensitive to nutrient intake and consequently BCS changes more drastically comparing to mature cows [7,11].

At the onset of the synchronization protocol, plasma concentrations of urea were higher for cows supplemented with blocks during postpartum. Blood urea nitrogen is traditionally a biological marker for crude protein (CP) or rumen degradable protein [6,39]. Studies have documented that the relationship between blood urea nitrogen and fertility in dairy cattle are negatively correlated (>19 mg/dL results in low fertility) [40]. Conversely, in beef cattle, urea concentration is not negatively associated with pregnancy risk [41], whereas optimal urea concentration in beef cows range from 10 to 25 mg/dL [39,41]. Our data showed that regardless of treatment, cows had urea concentration ranging from 10 to 16 mg/dL, which suggest that all the cows in the present study consumed adequate amounts of CP. The blocks supplements are highly palatable, which gives them the ability to mask undesirable flavors, such as urea and monensin [14,22]. Hence, this particularity of block supplements may be associated with higher urea concentrations 40 days postpartum for cows supplemented only in postpartum period.

Additionally, cows supplemented only in postpartum had greater glucose concentration at D80, which means better metabolic/nutritional status. Hence, the calves born by cows treated in postpartum were heavier at 120 days old. Notwithstanding, no CW differences at weaning were observed, probably because the supplementation ended at 120 days old and not at weaning (210 days old).

In conclusion, regardless of period of treatment, block supplementation increased BCS at parturition, pregnancy rate at first FTAI and overall pregnancy rate. Likewise, forty days postpartum, BW, BCS, RFAT, BFAT, and glucose concentration were greater for supplemented cows. Also, supplemented cows had greater insulin concentrations. Block supplementation during both pre and postpartum periods improved progeny growth until weaning. Under the conditions of the current experiment, block supplementation only during pre vs only during postpartum did not affect reproductive performance in primiparous cows. Block supplementation can be a tool to optimize fertility and calf performance in Nelore primiparous cows, facilitating nutritional management on farms.

## Figures and Tables

**Table 1:** Average chemical composition of supplements provided from day 90 to 120

Item	Supplement^1)^	

	C	B
DM (%)	92.5	90.0
TDN (%)	35.0	40.0
CP (%)	40.0	35.0
NPN (%)	5.1	3.5
Ca (g/kg)	80	65
P (g/kg)	30	20
Na (g/kg)	50	40
S (g/kg)	5	4
Zn (mg/kg)	1,400	920
Cu (mg/kg)	400	230
I (mg/kg)	20	13.5
Co (mg/kg)	22	17.5
Se (mg/kg)	7.2	9
Mn (mg/kg)	430	380
Sodium monensin (mg/kg)	-	300

DM, dry matter; TDN, total digestible nutrients; CP, crude protein; NPN, non protein nitrogen; Ca, calcium; P, phosphorous; Na, sodium; S, sulfur; Zn, zinc; Cu, copper; I, iodine; Co, cobalt; Se, selenium; Mn, manganese.

1) C, control supplementation offered daily at 0.06% of body weight/animal/d; B, Block supplementation offered weekly at 0.07% of body weight/animal/d.

**Table 2:** Herbage mass of pastures and average chemical composition of the green leaves sample collected during the experimental period

Item^2)^	Month^1)^			

	June	August	November	January
HM (kg DM/ha)	2.237	1.493	1.702	1.366
Green leaves (%)	10	29	53	55
CP (%)	14.8	14.3	8.8	9.6
Ca (%)	3.1	2.9	3.0	2.3
P (%)	2.1	1.5	2.1	1.6
Cu (mg/kg)	6.21	6.49	4.34	3.48
Mn (mg/kg)	82.66	77.63	52.26	32.53
Zn (mg/kg)	17.84	16.53	15.23	7.04

1) Forage samples were collected by hand plucked over the time before the grazing by females; June, begging of supplementation; August, prepartum period; November and January, postpartum period.

2) HM, herbage mass; Green leaves, percentage of green leaves on pasture; CP, crude protein; Ca, calcium; P, phosphorus; Cu, cooper; Mn, manganese; Zn, zinc.

**Table 3:** Effects of block supplementation during pre and/or postpartum on reproductive performance in primiparous Nelore cows

Variable (%, n/n)^1)^	Treatments^2)^				p-value^3)^		
	
	CC	CB	BC	BB	C1	C2	C3
Postpartum cyclicity rate at D40	15.7 (17/108)	18.8 (22/117)	18.4 (19/103)	22.5 (20/89)	0.39	0.51	0.97
Pregnancy rate at first FTAI	41.7 (45/108)	49.6 (58/117)	49.5 (51/103)	56.2 (50/89)	0.04	0.15	0.97
Pregnancy loss of first FTAI	8.9 (4/45)	8.6 (5/58)	5.9 (3/51)	10.0 (5/50)	0.98	0.30	0.60
Pregnancy rate at second FTAI	46.0 (29/63)	47.5 (28/59)	48.1 (25/52)	48.7 (19/39)	0.70	0.93	0.95
Pregnancy loss of second FTAI	3.4 (1/29)	7.1 (2/28)	4.0 (1/25)	5.3 (1/19)	0.98	0.97	0.63
Pregnancy rate at NM	35.9 (14/39)	49.5 (19/38)	41.9 (13/31)	53.8 (14/26)	0.28	0.72	0.51
Overall pregnancy rate at end of BS	76.9 (83/108)	83.8 (98/117)	82.5 (85/103)	86.5(77/89)	0.05	0.54	0.81

1) Cyclicity rate, presence of corpus luteum at the onset of the FTAI protocol (D40); FTAI, fixed-time artificial insemination; NM, natural mating; Overall pregnancy rate = 1st FTAI+2nd FTAI+NM; BS, breeding season.

2) CC: heifers received control supplement (C) in loose meal form (0.06% of body weight [BW] offered daily before and after parturition; n = 108); CB: received C before parturition and block supplementation (B) after parturition (0.07% of BW offered weekly after parturition; n = 117); BC: received B before and C after parturition (n = 103); BB: received B before and after parturition (n = 89).

3) Orthogonal contrasts: C1 (Block supplementation effect): Control (CC) vs block supplementation (BB+BC+CB); C2 (Block supplementation effect in both pre and postpartum periods): Pre and postpartum (BB) vs Pre or postpartum (BC+CB); and C3 (Pre or postpartum effect): prepartum (BC) vs postpartum (CB).

**Table 4:** Effect of prepartum and/or postpartum supplementation with blocks on body weight (kg) and body condition score (1–5 point scale) of primiparous Nelore cows evaluated at different times

Itens^1)^	Treatment^2)^				SEM	p-value^3)^				
	
	CC	CB	BC	BB		T	T×treat	C1	C2	C3
BW (kg)						<0.0001	<0.0001	0.83	0.98	0.76
90 days prepartuma	435.8	441.0	473.1	440.1	1.78	-	-	0.25	0.68	0.18
40 days postpartumb	400.8	412.2	400.9	413.3	1.70	-	-	0.03	0.16	0.001
120 days postpartumd	432.9	431.3	432.9	429.0	1.79	-	-	0.16	0.22	0.11
BCS, 1–5						<0.0001	0.002	0.05	0.03	0.60
90 days prepartum^4)^	2.91	2.95	2.93	2.94	0.01	-	-	0.15	0.45	0.64
Parturition	3.01	3.00	3.11	3.10	0.01	-	-	0.04	0.25	<0.001
40 days postpartum^5)^	2.83	2.90	2.90	2.95	0.02	-	-	0.02	0.21	0.99
80 days postpartum^6)^	2.84	2.91	2.85	2.98	0.02	-	-	0.12	0.02	0.12
120 days postpartum^7)^	3.00	3.07	2.99	3.04	0.02	-	-	0.56	0.65	0.20
170 days postpartum^8)^	2.78	2.87	2.74	2.84	0.01	-	-	0.34	0.33	0.001

SEM, standard error of the mean.

1) BW, body weight (kg); BCS, body condition score (1–5 point scale).

2) CC: heifers received control supplement (C) in loose meal form (0.06% of body weight [BW] offered daily before and after parturition; n = 108); CB: received C before parturition and block supplementation (B) after parturition (0.07% of BW offered weekly after parturition; n = 117); BC: received B before and C after parturition (n = 103); BB: received B before and after parturition (n = 89).

3) Orthogonal contrasts: C1 (Block supplementation effect): control (CC) vs block supplementation (BB+BC+CB); C2 (Block supplementation effect in both pre and postpartum periods): Pre and postpartum (BB) vs Pre or postpartum (BC+CB); and C3 (Pre or postpartum effect): prepartum (BC) vs postpartum (CB); T = time, days relative to calving; T×treat = interaction between sampling time and treatment.

4) 90 days prepartum = at the beginning of supplementation (D-90).

5) 40 days postpartum = at the onset of the synchronization protocol (D40).

6) 80 days postpartum = at pregnancy diagnosis and resynchronization (D80).

7) 120 days postpartum = at the end of supplementation and second pregnancy diagnosis (D120).

8) 170 days postpartum = at final pregnancy diagnosis after two FTAI’s and natural mating (D170).

**Table 5:** Effect of prepartum and/or postpartum supplementation for calf performance evaluated at 5 different times

Items	Treatment^1)^				SEM	p-value^2)^				
	
	CC	CB	BC	BB		T	Txtreat	C1	C2	C3
CW (kg)						<0.0001	<0.0001	0.11	0.20	0.13
Birth	34.6	35.4	35.7	35.6	0.65	-	-	0.01	0.89	0.16
80 days^3)^	97.8	98.7	99.7	106.4	0.78	-	-	0.03	<0.001	0.75
120 days^4)^	123.9	132.9	126.5	139.3	0.91	-	-	<0.001	<0.001	0.002
170 days^5)^	182.9	183.7	184.8	193.3	1.18	-	-	0.55	0.002	0.93
Weaning^6)^	207.7	208.8	210.0	216.2	1.45	-	-	0.38	0.02	0.74

SEM, standard error of the mean; CW, calf body weight.

1) CC: heifers received control supplement (C) in loose meal form (0.06% of body weight [BW] offered daily before and after parturition; n = 108); CB: received C before parturition and block supplementation (B) after parturition (0.07% of BW offered weekly after parturition; n = 117); BC: received B before and C after parturition (n = 103); BB: received B before and after parturition (n = 89).

2) Orthogonal contrasts: C1 (Block supplementation effect): Control (CC) vs block supplementation (BB+BC+CB); C2 (Block supplementation effect in both pre and postpartum periods): Pre and postpartum (BB) vs Pre or postpartum (BC+CB); and C3 (Pre or postpartum effect): prepartum (BC) vs postpartum (CB). T, time, days relative to calf age; T×treat, interaction between sampling time and treatment.

3) 80 days = calf body weight at 80 days old (D80).

4) 120 days = calf body weight at 120 days old and at the end of the supplementation period (D120).

5) 170 days = calf body weight at 170 days old (D170).

6) Weaning = calf body weight at 210 days old (D210).

**Table 6:** Effect of block supplementation during prepartum and/or postpartum on metabolic and hormone concentration in primiparous Nelore cows evaluated at different times

Items	Treatment^1)^				SEM	p-value^2)^				
	
	CC	CB	BC	BB		T	TreatxT	C1	C2	C3
Insulin (μg/mL)	**(10.80)**	**(13.88)**	**(12.39)**	**(12.55)**	0.49	<0.0001	0.91	0.008	0.13	0.26
90 days prepartum^3)^	13.75	14.94	14.21	14.11	0.78	-	-	-	-	-
40 days postpartum^4)^	6.66	9.49	9.93	8.63	0.58	-	-	-	-	-
80 days postpartum^5)^	12.10	17.11	13.25	15.04	1.05	-	-	-	-	-
IGF-1 (ng/mL)	**(388.5)**	**(405.8)**	**(423.8)**	**(418.7)**	11.16	<0.0001	0.79	0.24	0.35	0.99
90 days prepartum^3)^	390.6	388.7	401.3	384.6	7.57	-	-	-	-	-
40 days postpartum^4)^	260.7	265.4	265.7	273.1	7.59	-	-	-	-	-
80 days postpartum^5)^	526.9	569.4	613.0	607.4	20.70	-	-	-	-	-
Glucose (mg/dL)						0.20	0.0002	0.83	0.76	0.20
40 days postpartum^4)^	89.9	101.6	99.1	104.3	1.83	-	-	0.01	0.34	0.65
80 days postpartum^5)^	100.7	108.3	89.3	87.4	3.16	-	-	0.15	0.11	0.03
Urea (mg/dL)						0.009	0.07	0.04	0.19	0.22
40 days postpartum^4)^	10.31	16.24	10.81	15.03	0.70	-	-	0.03	0.33	0.004
80 days postpartum^5)^	13.34	14.40	14.57	14.44	0.51	-	-	0.41	0.97	0.91

The mean of the treatments is identified in parentheses.

SEM, standard error of the mean; IGF-1, insulin-like growth factor-1.

1) CC: heifers received control supplement (C) in loose meal form (0.06% of body weight [BW] offered daily before and after parturition; n = 108); CB: received C before parturition and block supplementation (B) after parturition (0.07% of BW offered weekly after parturition; n = 117); BC: received B before and C after parturition (n = 103); BB: received B before and after parturition (n = 89).

2) Orthogonal contrasts: C1 (Block supplementation effect): Control (CC) vs block supplementation (BB+BC+CB); C2 (Block supplementation effect in both pre and postpartum periods): Pre and postpartum (BB) vs Pre or postpartum (BC+CB); and C3 (Pre or postpartum effect): prepartum (BC) vs postpartum (CB).

3) 90 days prepartum= at the beginning of supplementation (D-90).

4) 40 days postpartum= at the onset of the synchronization protocol (D40).

5) 80 days postpartum= at pregnancy diagnosis and resynchronization (D80).

## References

[1] D’Occhio MJ, Baruselli PS, Campanile G (2019). Metabolic health, the metabolome and reproduction in female cattle: a review. Ital J Anim Sci.

[2] Wiltbank JN, Rowden WW, Ingalls JE, Geegoey KE, Koch RM (1962). Effect of energy level on reproductive phenomena of mature hereford cows. J Anim Sci.

[3] Santos FP, Dorea JR, Costa DFA, De Souza J, Batistel F (2014). Forage management and methods to improve nutrient intake in grazing cattle.

[4] De Gouvêa VN, Colli MHA, Gonçales WA (2018). The combination of β-carotene and vitamins improve the pregnancy rate at first fixed-time artificial insemination in grazing beef cows. Livest Sci.

[5] Stobbs TH (1975). Factors limiting the nutritional value of grazed tropical pastures for beef and milk production. Trop Grasslands.

[6] Sotelo D, Paulino MF, Rennó LN (2018). Performance and metabolic status of grazing beef heifers receiving increasing protein supplementation pre- and postpartum. Anim Prod Sci.

[7] Spitzer JC, Morrison DG, Wettemann RP, Faulkner LC (1995). Reproductive responses and calf birth and weaning weights as affected by body condition at parturition and postpartum weight gain in primiparous beef cows. J Anim Sci.

[8] Hess BW, Lake SL, Scholljegerdes EJ (2005). Nutritional controls of beef cow reproduction. J Anim Sci.

[9] Ayres H, Ferreira RM, Torres-Júnior JRS (2014). Inferences of body energy reserves on conception rate of suckled Zebu beef cows subjected to timed artificial insemination followed by natural mating. Theriogenology.

[10] Wettemann RP, Lents CA, Ciccioli NH, White FJ, Rubio I (2003). Nutritional- and suckling-mediated anovulation in beef cows. J Anim Sci.

[11] De Moura FH, Costa TC, Trece AS (2020). Effects of energy-protein supplementation frequency on performance of primiparous grazing beef cows during pre and postpartum. Asian-Australas J Anim Sci.

[12] Sales JNS, Bottino MP, Silva LACL (2016). Effects of eCG are more pronounced in primiparous than multiparous Bos indicus cows submitted to a timed artificial insemination protocol. Theriogenology.

[13] DelCurto T, Hess BW, Huston JE, Olson KC (2000). Optimum supplementation strategies for beef cattle consuming low-quality roughages in the western United States. J Anim Sci.

[14] Kunkle WE, Johns JT, Poore MH, Herd DB (2000). Designing supplementation programs for beef cattle fed forage-based diets. J Anim Sci.

[15] Meteer WC, Shike DW, De Cardoso FC (2015). Prepartum and postpartum nutritional management to optimize fertility in beef cattle. Acta Sci Vet.

[16] D’Occhio MJ, Baruselli PS, Campanile G (2019). Influence of nutrition, body condition, and metabolic status on reproduction in female beef cattle: a review. Theriogenology.

[17] Bailey CR, Goetsch AL, Hubbell DS, Rosenkrans CF (2008). Effects of monensin on beef cow reproduction. Can J Anim Sci.

[18] Hardin DR, Randel RD (1983). Effect of monensin on postpartum interval to first estrus and serum LH response to 0, 1, 2 or 4 mg estradiol-17β at 21 days postpartum. Theriogenology.

[19] Cassini MH, Hermitte G (1992). Patterns of environmental use by cattle and consumption of supplemental food blocks. Appl Anim Behav Sci.

[20] Bailey DW, Welling GR (2007). Evaluation of low-moisture blocks and conventional dry mixes for supplementing minerals and modifying cattle grazing patterns. Rangel Ecol Manag.

[21] Löest CA, Titgemeyer EC, Drouillard JS, Lambert BD, Trater AM (2001). Urea and biuret as nonprotein nitrogen sources in cooked molasses blocks for steers fed prairie hay. Anim Feed Sci Technol.

[22] Moriel P, Vendramini JMB, Carnelos C, Piccolo MB, da Silva HM (2019). Effects of monensin on growth performance of beef heifers consuming warm-season perennial grass and supplemented with sugarcane molasses. Trop Anim Health Prod.

[23] Valadares Filho S, de C, Silva LFCe, Gionbelli MP, Gráfica Suprema (2016). Nutrient requirements of Zebu and crossbred cattle - BR-CORTE. Exig Nutr Zebuínos Puros e Cruzados - BR-CORTE, Editora Federal de Viçosa.

[24] Ayres H, Ferreira RM, de Souza Torres JR, Demétrio CGB, de Lima CG, Baruselli PS (2009). Validation of body condition score as a predictor of subcutaneous fat in Nelore (Bos indicus) cows. Livest Sci.

[25] Shoup LM, Kloth AC, Wilson TB (2015). Prepartum supplement level and age at weaning: I. Effects on pre- and postpartum beef cow performance and calf performance through weaning. J Anim Sci.

[26] Vizcarra JA, Wettemann RP, Spitzer JC, Morrison DG (1998). Body condition at parturition and postpartum weight gain influence luteal activity and concentrations of glucose, insulin, and nonesterified fatty acids in plasma of primiparous beef cows. J Anim Sci.

[27] Williams AR (2002). Ultrasound applications in beef cattle carcass research and management. J Anim Sci.

[28] Marques RS, Cooke RF, Rodrigues MC, Moriel P, Bohnert DW (2016). Impacts of cow body condition score during gestation on weaning performance of the offspring. Livest Sci.

[29] Schelling GT (1984). Monensin mode of action in the rumen. J Anim Sci.

[30] Reed BK, Whisnant CS (2001). Effects of monensin and forage: concentrate ratio on feed intake, endocrine, and ovarian function in beef heifers. Anim Reprod Sci.

[31] Matos M (2004). Monensin supplementation pre and postpartum associated synchronization of the ovulation protocols in nelore cows. Dissertation.

[32] Laskowski D, Sjunnesson Y, Humblot P, Andersson G, Gustafsson H, Båge R (2016). The functional role of insulin in fertility and embryonic development-What can we learn from the bovine model?. Theriogenology.

[33] Wettemann RP, Bossis I (2000). Energy intake regulates ovarian function in beef cattle. J Anim Sci.

[34] Sá Filho MF, Penteado L, Reis EL, Reis TANPS, Galvão KN, Baruselli PS (2013). Timed artificial insemination early in the breeding season improves the reproductive performance of suckled beef cows. Theriogenology.

[35] Baruselli PS, Ferreira RM, Sá Filho MF, Bó GA (2018). Review: Using artificial insemination v. natural service in beef herds. Animal.

[36] Freitas BG, Mingoti RD, Monteiro BM (2021). Relationship of body maturation with response to estrus synchronization and fixed-time AI in Nelore (Bos indicus) heifers. Livest Sci.

[37] Callaghan MJ, Rodgers RJ, Perry VEA (2020). Supplementation of rangeland primiparous Bos indicus x Bos taurus beef heifers during lactation. 1. Effects on dam milk production and liveweight, bull calf growth, live carcass characteristics and metabolic hormone concentrations. Theriogenology.

[38] Bohnert DW, Stalker LA, Mills RR, Nyman A, Falck SJ, Cooke RF (2013). Late gestation supplementation of beef cows differing in body condition score: Effects on cow and calf performance. J Anim Sci.

[39] Hill SL, Olson KC, Jaeger JR, Stevenson JS (2018). Serum and plasma metabolites associated with postpartum ovulation and pregnancy risks in suckled beef cows subjected to artificial insemination. J Anim Sci.

[40] Rhoads ML, Rhoads RP, Gilbert RO, Toole R, Butler WR (2006). Detrimental effects of high plasma urea nitrogen levels on viability of embryos from lactating dairy cows. Anim Reprod Sci.

[41] Gunn PJ, Lundberg AL, Cushman RA, Freetly HC, Amundson OL Effect of circulating blood or plasma urea nitrogen concentrations on reproductive efficiency in beef heifers and cows. Iowa State University Animal Industry Report 13. Animal Industry Report.

